# Sustained Interleukin-1*β* Exposure Modulates Multiple Steps in Glucocorticoid Receptor Signaling, Promoting Split-Resistance to the Transactivation of Prominent Anti-Inflammatory Genes by Glucocorticoids

**DOI:** 10.1155/2015/347965

**Published:** 2015-04-22

**Authors:** Pedro Escoll, Ismael Ranz, Norman Muñoz-Antón, Ana van-den-Rym, Melchor Alvarez-Mon, Carlos Martínez-Alonso, Eva Sanz, Antonio de-la-Hera

**Affiliations:** ^1^Department of Medicine, School of Medicine, Alcala University (UAH), Alcalá de Henares, 28805 Madrid, Spain; ^2^Biologie des Bactéries Intracellulaires, Departement Genomes et Génétique, Institut Pasteur, 75015 Paris, France; ^3^Immunology and Individualized Medicine, IMMPA CSIC/UAH Joint Unit, Alcalá de Henares, 28805 Madrid, Spain; ^4^MRC & Asthma UK Centre in Allergic Mechanisms of Asthma, King's College London, Division of Asthma, Allergy & Lung Biology, 5th Floor Tower Wing, Guy's Hospital, London SE1 9RT, UK; ^5^Department of Immunology and Oncology, National Biotechnology Center, Spanish National Research Council (CNB-CSIC), 28049 Madrid, Spain

## Abstract

Clinical treatment with glucocorticoids (GC) can be complicated by cytokine-induced glucocorticoid low-responsiveness (GC-resistance, GCR), a condition associated with a homogeneous reduction in the expression of GC-receptor- (GR-) driven anti-inflammatory genes. However, GR level and phosphorylation changes modify the expression of individual GR-responsive genes differently. As sustained IL-1*β* exposure is key in the pathogenesis of several major diseases with prevalent GCR, we examined GR signaling and the mRNA expression of six GR-driven genes in cells cultured in IL-1*β* and afterwards challenged with GC. After a GC challenge, sustained IL-1*β* exposure reduced the cytoplasmic GR level, GR^Ser203^ and GR^Ser211^ phosphorylation, and GR nuclear translocation and led to selective GCR in the expression of the studied genes. Compared to GC alone, in a broad range of GC doses plus sustained IL-1*β*, FKBP51 mRNA expression was reduced by 1/3, TTP by 2/3, and IRF8 was completely knocked down. In contrast, high GC doses did not change the expression of GILZ and DUSP1, while IGFBP1 was increased by 5-fold. These effects were cytokine-selective, IL-1*β* dose- and IL-1R1-dependent. The integrated gain and loss of gene functions in the “split GCR” model may provide target cells with a survival advantage by conferring resistance to apoptosis, chemotherapy, and GC.

## 1. Introduction

Cortisol, a steroid of the glucocorticoid (GC) class, is a major hormone with several physiological roles in modulating the immune response and the cell survival/apoptosis balance. These functions provided a rationale to develop synthetic GCs, such as dexamethasone (DEX), which are mainstay drugs to treat inflammation and cancer [1–8]. However, groups of patients display acquired low responsiveness to GC, a condition termed GC-resistance (GCR). Partial or complete GCR causes treatment inefficacy, which forces clinicians to raise the GC therapeutic dose and can lead to either detrimental side effects or GC withdrawal [1–8]. The development of GCR depends on the cytokine environment in an individual patient's lesion and also varies among diseases [1, 2, 5].

Interleukin- (IL-) 1*β* was not originally cited among major GCR-inducing cytokines [1–8], but sustained exposure to IL-1*β* is key in the pathogenesis of several major diseases with prevalent GCR [4, 9–21]. Importantly, IL-1*β* triggers secretory cascades including several molecules that promote GCR (e.g., IL-2, IL-6, IL-17, GM-CSF, and TNF-*α*) [1, 4, 6–8, 22–24]. Current GCR models propose an interplay between cytokines and the GC receptor (GR) [2, 23–28]: GC-activated GR downregulates inflammation, but sustained exposure to cytokines before the GC treatment overrides GC actions, which can lead to GCR, reinforced inflammation, and increased cell survival by blockade of apoptosis [1–8, 29–32].

Both GC and IL-1*β* signaling are tightly regulated, and they have several natural crosstalk nodes. IL-1*β* neuroendocrine activity triggers GC secretion, which activates negative feedback loops [2, 9, 11, 13, 15, 17, 18, 33]. Receptors that trigger IL-1*β* production also activate cell-autonomous signals that downregulate proinflammatory gene expression (e.g., via the suppression of MAPK by dual-specific phosphatase 1, DUSP1) and promote IL-10 and interferon (IFN) secretion [2, 22–24, 34–36]. DUSP1, IL-10, and IFN downregulate IL-1*β* responses by different mechanisms including upregulation of tristetraprolin (TTP, a mRNA-binding protein that degrades proinflammatory mRNAs) and apoptosis inhibitory proteins such as glucocorticoid-induced leucine zipper (GILZ, a NF-*κ*B inhibitor) and IFN-response factor (IRF-) 8 [2, 3, 23–28]. Notably, TTP, DUSP1, GILZ, and IRF8 expression is also directly activated by GR-driven transcription; these genes are responsible for many of the effects of GC [1–3, 29–32, 37]. Different cytokines are proposed to “generally” compromise GC transactivation potential of these genes, which would result in a “global” block of endogenous GR-driven suppression of inflammation [2, 3, 32, 33, 37–41]. Since patients suffer inflammation and cytokine exposure before GC treatments, sustained proinflammatory conditions are mimicked* in vivo* and* in vitro* by cytokine preincubation and cytokine + GC challenges [2]. While sustained IL-1R1 signaling, after prolonged incubation with either IL-1*α* or IL-1*β*, is known to reduce GR nuclear translocation and promote GCR, its effects on GC transactivation of endogenous anti-inflammatory genes have not been investigated [2, 34–36, 42].

GCs act by binding to cytoplasmic GR, which is followed by GR Ser^203^ phosphorylation (p-GR^Ser203^) and the exchange of FKBP51 for FKBP52 in the GC/GR-chaperone containing complex. The complex then translocates to the nucleus, where it is active as a transcription factor [2, 3, 38, 41]. GCs increase FKBP51 mRNA expression proportionally to the amount of nuclear GC/GR, making the endogenous FKBP51 level an* in vivo* biomarker of GC-responsiveness and GCR [2, 33, 37]. The expression of some GC-driven genes is strongly dependent on p-GR^Ser211^, which recruits distinct GR transcription cofactor combinations. These p-GR^Ser211^ effects are target gene-specific and in some cases also depend on the level of nuclear GC/GR, shown by comparing the effect of p-GR^Ser211^ activation on relative GILZ, IRF8, and insulin growth factor binding protein (IGFBP1) mRNA expression levels [2, 3, 32, 37–41, 43]. Some mechanisms of inflammation modify the GR phosphorylation profile in a GC-independent manner, which promotes selective modifications in the patterns of GR-driven target gene expression and has been suggested to cause GR to signal differently in disease compared to healthy cells. However, cytokine effects in this novel GCR model were not addressed [42]. The A549 lung cancer epithelial cell line is among the cell systems that were instrumental to define the current GR signaling model and responds naturally to IL-1*β* [2, 38, 41]. This line was therefore selected for this study to examine the interaction between IL-1*β* and GR signaling.

Recent GCR models propose that sustained cytokine exposure generally compromises GC transactivation potential of endogenous GR-driven genes, which results in a uniform/global loss of anti-inflammatory functions [2, 33, 44, 45]. However, given that the genome-wide determination of the GC response has revealed an unexpected specificity in GR-driven expression of individual genes [32, 37–41, 43, 46] and that cellular stress conditions associated with inflammation modify the GR-driven gene expression pattern [1–3, 42], we hypothesized that the sustained exposure of A549 cells to IL-1*β* may differentially affect the mRNA expression of individual DEX/GR-driven endogenous genes.

## 2. Materials and Methods

### 2.1. Cells and Reagents

A549 cells (ref. CCL-185, American Type Culture Collection ATCC, Rockville, MD) were cultured in phenol-red-free Dulbecco's modified Eagle's medium (Lonza, Walkersville, MD) supplemented with 10% (v/v) charcoal/dextran-treated fetal bovine serum to avoid steroid activity (Hyclone, Logan, UT). Dexamethasone was from Calbiochem (Nottingham, UK). Recombinant human IL-1*β* and IL-1ra were from R&D (Abingdon, UK), and recombinant human IL-18 was from MBL International (Nagoya, Japan). Stock solutions of the reagents were dissolved in either ethanol (DEX) or PBS supplemented with 0.1% human serum albumin (IL-1*β*, IL-1ra, and IL-18), and working solutions were diluted in complete medium before addition to cell cultures.

### 2.2. Western Blots

The NE-PER extraction kit (Pierce Biotechnology, Erembodegem, Belgium) was used according to the manufacturer's instructions to obtain nuclear and cytoplasmic protein extracts. Western blots followed standard protocols [16, 38]. The total protein concentration in each lysate was measured to normalize the amount loaded in the gels. Antibodies against *β*-actin, *α*-tubulin, or p53 were used as references in each lane for the relative quantifications of the indicated targets. The sources of the antibodies specific against the human antigens were GR (BD Biosciences San Diego, CA); *β*-actin, p53, *α*-tubulin, p-GR^Ser211^ (Cell Signaling Technology, Danvers, MA); and p-GR^Ser203^ (ab79268, Abcam, Cambridge, UK). The use of IRDye-800CW and IRDye-680-labeled secondary antibodies (Li-Cor, Lincoln, NE) allowed simultaneous quantitation of target and reference antigens with the Odyssey infrared digital imaging system (Li-Cor).

### 2.3. Immunofluorescence, High Content Analysis (HCA) Microscopy, and Cell Shape Measurements

Cells were cultured in 24-well plates over 12 mm-diameter round coverslips (Menzel-Gläser, Braunschweig, Germany) coated with 5 *μ*g/cm^2^ poly-D-lysine (BD Biosciences) for the indicated time periods, then washed, and fixed for 20 min in methanol chilled to −20°C and permeabilized for 30 sec in acetone (Merck, Darmstadt, Germany). The primary antibodies to human antigens were GR (Clone 57, Affinity Bioreagents, Golden, CO), p-Ser^211^-GR, and *β*-actin (Cell Signaling Technology). Secondary AlexaFluor488- and Texas-Red-conjugated antibodies were from Molecular Probes (Life Technologies, Foster City, CA, USA). The coverslips were mounted with DAPI and antifading solution (10 mg/mL orthophenyendiamine, 90% glycerol, pH 8). High content analysis (HCA) was performed in a Scan^∧^R automated microscope station (Olympus, Hamburg, Germany), and nuclear (DAPI) and cytoplasmic (Texas-Red) staining areas were quantified for GR intensity (AlexaFluor488) by Scan^∧^R software, with at least 2500 cells per slide analyzed in each compartment [2, 3, 44, 45]. To measure the area and circularity in individual cells, the Scan^∧^R phase contrast images were processed using NIH ImageJ 2.0 software (Bethesda, MD). Briefly, circularity was measured using the formula 4*π*∗area/perimeter^2^. A value of 1.0 indicates a perfect circle. As the value approaches 0.0, it indicates an increasingly elongated shape [2, 3, 46].

### 2.4. RT-qPCR

RNA was reverse transcribed with the high capacity cDNA reverse transcription kit, and cDNA was quantitated by PCR using a 7900HT real-time PCR system and specific Taqman assays for GR-*α*, GR-*β*, FKBP51, TTP, DUSP-1 (formerly MKP1), GILZ, and IRF8, normalized with an endogenous control (*β*-actin), using the 2^−ΔΔCt^ method, all according to the manufacturer's instructions (all from Life Technologies).

### 2.5. Statistical Analysis

The results are expressed as mean ± SEM or mean ± SD as specified in the figure legends and were statistically tested using the unpaired two-tailed *t*-test or one-way ANOVA, followed by the Tukey-Kramer multiple comparison test using Prism v5 (GraphPad Software, La Jolla, CA). Results were considered significant when *P* < 0.05. Nonlinear regression analysis showed 95% confidence intervals.

## 3. Results and Discussion

### 3.1. Sustained IL-1*β* Conditions Inhibit GC-Promoted GR Nuclear Translocation

**Figure 1:**
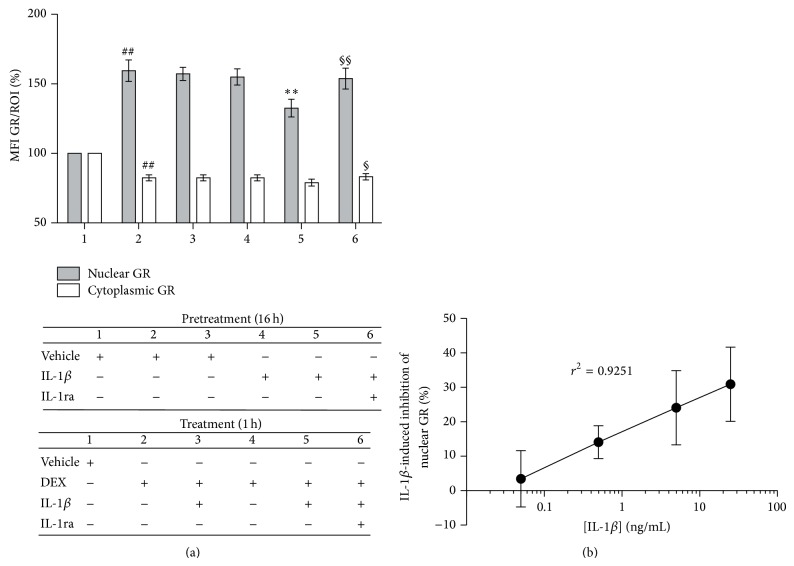
Sustained IL-1*β* conditions reduce DEX-driven nuclear GR translocation in a dose-dependent manner, mediated by IL-1R1. Cells were pretreated with either vehicle (veh) or 5 ng/mL IL-1*β* for 16 h and then treated for 1 h with either veh, 10^−8^ M DEX or 5 ng/mL IL-1*β* + 10^−8^ M DEX, in the absence or presence of 1 *μ*g/mL IL-1ra, as detailed in the table below the histogram. After culture, the cells were fixed, permeabilized, and stained with *β*-actin- and GR-specific antibodies. They were then labeled with secondary fluorescent antibodies and DAPI, mounted, and subjected to HCA three-color fluorescence microscopy. *β*-actin and DAPI staining were used to demarcate the cytoplasmic and nuclear regions of interest (ROI), respectively. (a) Histogram bars represent the HCA results for each culture condition, numbered 1–6. Changes in the levels of nuclear (gray column) and cytoplasmic (white column) GR are expressed as % mean fluorescence intensity (MFI)/nuclear or cytoplasmic ROI ± SD (*n* = 5), relative to GR content in vehicle-treated cells, which are set as 100%. ^##^ *P* < 0.005, differences in either nuclear or cytoplasmic GR between veh/DEX-treated (a2) and veh/veh-treated cells (a1). ^∗∗^ *P* < 0.005, difference in nuclear GR between IL-1*β*/IL-1*β* + DEX-treated (a5) and DEX-treated cells (a2). ^§§^ *P* < 0.005 and ^§^ *P* < 0.05, differences between IL-1*β* + IL-1ra/IL-1*β* + IL-1ra + DEX-treated (a6) and IL-1*β*/IL-1*β* + DEX-treated cells (a5) in their nuclear or cytoplasmic GR level, respectively. (b) Dose-dependent inhibition of nuclear GR translocation was assayed in cells pretreated with titrated concentrations of IL-1*β* (50 pg/mL to 25 ng/mL) for 16 h and then treated for 1 h with the corresponding IL-1*β* concentration +10^−8^ M DEX. The results show the mean percentage of inhibition of GR nuclear translocation observed under sustained IL-1*β* conditions, compared to veh/10^−8^ M DEX-alone conditions and their 95% confidence intervals.

To determine whether sustained IL-1*β* exposure downregulates GR signaling, potentially leading to GCR, we first compared the effects of distinct IL-1*β* culture conditions on DEX-promoted GR nuclear translocation. We quantified cytoplasmic and nuclear GR protein in large numbers of cells by GR-immunofluorescence staining and HCA microscopy (Figure 1 and Figure  S1 in Supplementary Material available online at http://dx.doi.org/10.1155/2015/347965). DEX alone promoted significant increases in nuclear GR and significant reductions in cytoplasmic GR (Figure 1(a)(2); ^##^ *P* < 0.005), as expected [1–3, 60]. The rationale for the selected DEX dose (10^−8^ M = 10 nM) was to use the same dose that has been uniformly reported in studies of several GR signaling pathway mechanisms and allows for cross-comparison of our results [30, 32, 35, 37, 40, 61]. Under sustained IL-1*β* conditions (16 h preincubation with IL-1*β* plus 1 h coincubation with DEX and IL-1*β*), GR nuclear levels were significantly lower than those induced by DEX (Figure 1(a)(5); ^∗∗^ *P* < 0.005, averaging a 28.1 ± 3.7% reduction compared to DEX alone), which was not accompanied by the expected reciprocal increase in cytoplasmic GR. Importantly, neither a 1 h IL-1*β* coincubation with DEX nor 16 h IL-1*β* preincubation before a 1 h DEX-alone challenge caused significant changes in nuclear or cytoplasmic GR levels compared to DEX alone (Figures 1(a)(3) and 1(a)(4), resp.), indicating that sustained IL-1*β* exposure is required for IL-1*β* to inhibit DEX-induced nuclear GR translocation. These results were confirmed by Western blot and quantitative densitometry (Figure S2). Under sustained IL-1*β* conditions, the DEX-induced increase in nuclear GR protein level fell significantly, by 33.2 ± 4.8% (16 h preincubation with IL-1*β* + 2 h coincubation with DEX + IL-1*β*, Figure S2B-3; ^∗∗^ *P* < 0.005, compared to 2 h DEX alone), which was not accompanied by significant reciprocal increases in cytoplasmic GR compared to DEX alone (Figure S2B-6).

To determine whether the IL-1*β* inhibition of DEX-driven GR nuclear translocation was dose-dependent, we repeated the experiment in Figure 1(a) with titrated amounts of IL-1*β* (50 pg/mL to 25 ng/mL; Figure 1(b)). IL-1*β*-driven reductions in nuclear GR levels indeed displayed a linear dose-dependent response (*r* ^2^ = 0.9251). Together, these results demonstrate that sustained IL-1*β* conditions are required for this signal to significantly reduce, in a dose-dependent manner, the nuclear GR translocation promoted by DEX. In addition, the data demonstrate that neither a 1 h IL-1*β* coincubation with DEX nor a 16 h IL-1*β* preincubation before a 1 h DEX-alone challenge caused significant changes in nuclear or cytoplasmic GR levels compared to DEX alone. It supports the notion that the reported change in GR signaling selectively occurs under sustained IL-1*β* conditions (IL-1*β*/IL-1*β* + DEX), after a direct comparison of the above conditions, which has not been previously reported [34, 35].

We next asked whether these effects of sustained IL-1*β* are mediated by IL-1R1 (Figure 1(a)(6)). Quantitative analyses showed that a natural selective IL-1R1 antagonist, IL-1ra [2, 3, 16], inhibits the effects of sustained IL-1*β*: both nuclear (^§§^ *P* < 0.005) and cytoplasmic (^§^ *P* < 0.05) GR levels were significantly higher under sustained IL-1*β* conditions in the presence of IL-1ra (Figure 1(a)(6)). After addition of IL-1ra, the levels of nuclear and cytoplasmic GR were comparable to those observed in the absence of IL-1*β* (nonsignificant differences compared to DEX alone, Figure 1(a)(6)). These findings demonstrate that the inhibition of GR nuclear translocation by sustained IL-1*β* treatment is dependent on IL-1R1.

### 3.2. IL-1*β* Reduces Cytoplasmic GR Levels

The lack of a significant reciprocal increase in cytoplasmic GR levels suggested an additional reduction in the total GR protein amount (GR^total^), which we further investigated. As GC ligand-dependent signals transiently downregulate GR expression at the mRNA and protein levels [2, 3, 35], we tested whether sustained IL-1*β* treatment affected GR at either level. We first studied whether compared to DEX alone sustained IL-1*β* conditions modified the mRNA expression of GR-*α* or GR-*β* (Figure S3). GR-*α* and GR-*β* are splice variants with distinct functions transcribed from the GR/NR3C1 gene [2, 3, 62]. DEX alone downregulated both GR-*α* and GR-*β* mRNA in A549 cells, consistent with previous results in this cell line when treated with the GC budesonide [33, 41, 43, 60, 63]. Notably, sustained IL-1*β* conditions did not significantly change the DEX-induced GR-*α* and GR-*β* mRNA downregulation (Figure S3, nonsignificant differences), suggesting that modification of GR mRNA expression does not directly explain the effects of IL-1*β*.

**Figure 2:**
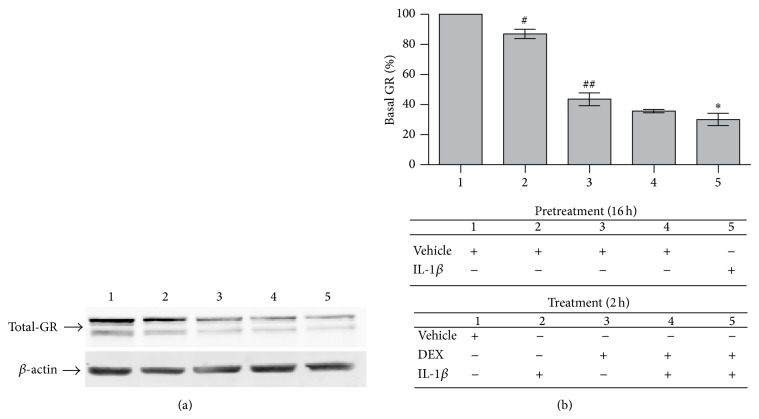
Sustained IL-1*β* reduces the whole-cell GR protein level after a DEX challenge. Cells were pretreated for 16 h with either vehicle or 5 ng/mL IL-1*β* and then treated for 2 h with vehicle (b1, veh/veh), 5 ng/mL IL-1*β* alone (b2, veh/IL-1*β*), 10^−8^ M DEX alone (b3, veh/DEX), simultaneous addition of 5 ng/mL IL-1*β* + 10^−8^ M DEX (b4, veh/IL-1*β* + DEX) or sustained IL-1*β* conditions (b5, IL-1*β*/IL-1*β* + DEX), as indicated in the table. Whole cell lysates were prepared and analyzed by Western blot with anti-GR and -*β*-actin antibodies. (a) Blots of a representative experiment (*n* = 3). (b) Quantitation of whole cell GR protein by densitometry. Data are represented as mean ± SEM percentage of total GR protein amount as compared to veh/veh treatment. ^#^ *P* < 0.05 and ^##^ *P* < 0.005, difference in GR protein levels between veh/IL-1*β*-treated (b2) or veh/DEX-treated (b3) and veh/veh-treated cells (b1), respectively. ^∗^ *P* < 0.05, difference in GR protein levels between IL-1*β*/IL-1*β* + DEX-treated (b5) and veh/DEX-treated cells (b3).

However, GR and *β*-actin immunoblots indicated that GR^total^ is decreased in cells treated with IL-1*β*-alone (Figure 2(a)). In the corresponding densitometry analyses of GR levels in whole cell lysates (Figure 2(b)), 2 h treatment with IL-1*β* alone significantly decreased GR^total^ by an average of 13.1 ± 1.9% (Figure 2(b)(2); ^#^ *P* < 0.05), whereas 2 h with DEX alone reduced GR^total^ by 56.4 ± 5.1% (Figure 2(b)(3); ^##^ *P* < 0.005). A 2 h IL-1*β* + DEX coincubation showed a mild additive reduction in GR^total^ levels, by 64.3 ± 1.1%, a nonsignificant decrease compared to DEX alone (Figure 2(b)(4)). Sustained IL-1*β*, however, significantly potentiated GR depletion by DEX, with GR^total^ reduced by 70.0 ± 5.3% (16 h preincubation with IL-1*β* + 2 h coincubation with DEX + IL-1*β*, Figure 2(b)(5); ^∗^ *P* < 0.05, compared to 2 h DEX-alone). The reduction in GR^total^ levels after sustained IL-1*β* conditions is mediated by IL-1R1, as they are abrogated by IL-1ra: IL-1ra reverts GR^total^ to levels similar to DEX alone (Figure S4).

**Figure 3:**
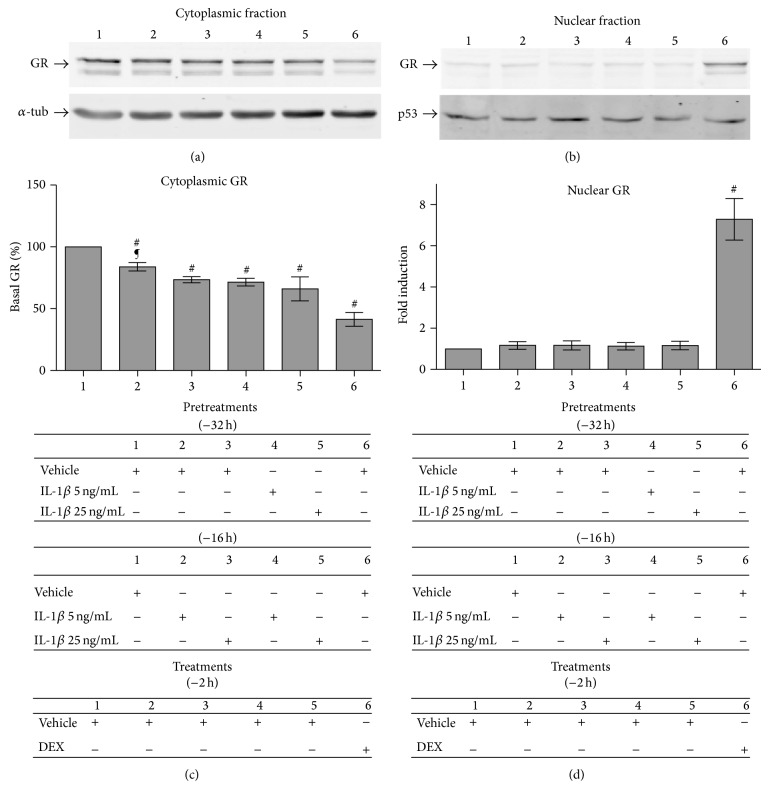
IL-1*β* alone reduces cytoplasmic GR protein levels without promoting GR nuclear translocation. Cells were pretreated at −32 h and −16 h with vehicle, 5 or 25 ng/mL IL-1*β*, and then treated the last 2 h with either vehicle or 10^−8^ M DEX alone, as indicated in the table: 1, 32 h in vehicle; 2, 16 h veh/16 h 5 ng/mL IL-1*β*; 3, 16 h veh/16 h 25 ng/mL IL-1*β*; 4, 32 h 5 ng/mL IL-1*β*; 5, 32 h 25 ng/mL IL-1*β*; and 6, 30 h veh/2 h 10^−8^ M DEX. The nuclear and cytoplasmic compartments were then fractionated and their lysates studied by Western blot with anti-GR antibody. Anti-*α*-tubulin (*α*-tub) and -p53 antibodies are controls for the correct cytoplasmic (a) and nuclear (b) compartment fractionation, respectively, in the representative blots (*n* = 3). Quantitation of cytoplasmic (c) and nuclear (d) GR protein by densitometry. Data are represented as mean ± SEM. ^#^ *P* < 0.05, differences in cytoplasmic GR protein content between cells treated with 16 h veh/16 h 5 ng/mL IL-1*β* (c2), 16 h veh/16 h 25 ng/mL IL-1*β* (c3), 32 h 5 ng/mL IL-1*β* (c4), 32 h 25 ng/mL IL-1*β* (c5), or 30 h veh/2 h DEX (c6), compared with 32 h vehicle-treated cells (c1). ^¶^ *P* < 0.05, difference in cytoplasmic GR protein levels between cells treated with 5 ng/mL (c2) and 25 ng/mL (c3) IL-1*β* for the last 16 h. ^#^ *P* < 0.05, difference in nuclear GR protein levels between 30 h veh/2 h DEX-treated (d6) and veh-treated cells (d1).

While GC-induced GR-protein degradation is a negative feedback loop that limits GC responsiveness and has a recognized role in GCR [2, 3], we are not aware of any report of IL-1*β*-driven GR protein downregulation. On the contrary, it has been proposed that, in mouse fibroblasts, GR^total^ increases after IL-1*α* exposure [2, 3, 34, 35, 38–41], and, in human airway muscle cells, cytokines induce nuclear GR translocation in a “nonspecific” “unliganded” (GC-independent) manner without reducing GR^total^ [2, 3, 38–41, 62]. To resolve this discrepancy, we next addressed whether the GR reductions induced by IL-1*β* alone occur in the cytoplasmic and/or nuclear compartments and whether IL-1*β* alone promotes significant “unliganded” GR nuclear translocation. We performed immunoblots and the corresponding GR densitometry in the nuclear and cytoplasmic fractions of cells incubated with either vehicle, IL-1*β* alone or DEX alone (Figure 3). The purity of the loaded nuclear and cytoplasmic cell fractions was assessed by p53 and *α*-tubulin codetection, respectively. The decrease in GR^total^ protein driven by IL-1*β* alone was linked to significant reductions in cytoplasmic but not nuclear GR (lanes 2–5), compared to vehicle (lane 1). Further, compared to DEX alone (lane 6), IL-1*β* alone did not drive any significant GR nuclear translocation.

The reductions in cytoplasmic GR driven by the cytokine were related to the IL-1*β* incubation time and dose. Quantitative analyses showed that increasing the time of IL-1*β* exposure further reduced the cytoplasmic GR level: 16 h and 32 h incubations in 25 ng/mL IL-1*β* significantly decreased cytoplasmic GR by an average of 26.6 ± 2.2% and 34.0 ± 7.3%, respectively (Figure 3(c)(3); ^#^ *P* < 0.05 and Figure 3(c)(5); ^#^ *P* < 0.05, IL-1*β* compared to vehicle). Lower doses of IL-1*β* (5 ng/mL) significantly reduced cytoplasmic GR by 16.2 ± 2.9% and 30.6 ± 1.1% after 16 h and 32 h, respectively (Figure 3(c)(2); ^#^ *P* < 0.05 and Figure 3(c)(4); ^#^ *P* < 0.05, IL-1*β* compared to vehicle). Notably, 5 ng/mL IL-1*β* was significantly less effective than 25 ng/mL IL-1*β* at 16 h (Figure 3(c)(2); ^¶^ *P* < 0.05) but not at 32 h, when the IL-1*β*-alone-driven cytoplasmic GR reduction tended to reach a dose plateau (Figure 3(c)(4), nonsignificant differences). DEX alone was more potent and reduced the cytoplasmic GR more quickly, by 58.6 ± 4.2%, than IL-1*β* alone (Figure 3(c)(6); ^#^ *P* < 0.05; 2 h DEX-alone compared to any IL-1*β*-alone condition). Together, these results suggest that the levels of reduction in cytoplasmic GR promoted by IL-1*β* could affect GC-responsiveness under IL-1*β* conditions, given that similar total GR level changes before GC challenges have been shown to determine the potency of subsequent anti-inflammatory GR-driven responses and their gene profile specificity [33, 38–41, 43, 63].

IL-1*β* alone did not promote any detectable increase in nuclear GR compared to vehicle (nonsignificant differences; IL-1*β* alone induced 0.11 to 0.17-fold increases over vehicle, Figure 3(d)(1–5)). As expected [2, 3, 38], 2 h DEX-alone promoted significant GR nuclear translocation (7.3-fold increase in nuclear GR levels over vehicle; Figure 3(d)(6); ^#^ *P* < 0.05 compared to vehicle or any of the IL-1*β*-alone incubations). To confirm that prolonged exposure to IL-1*β* alone reduces GR levels without promoting statistically significant unliganded GR nuclear translocation, we also carried out GR immunofluorescence and HCA microscopy assays, quantifying GR protein levels in the nucleus and cytoplasm (Figure S5). A 16 h IL-1*β* preincubation + 1 h IL-1*β*-alone challenge significantly reduced both nuclear and cytoplasmic GR levels (Figure S5-4; # for both nuclear and cytoplasmic GR, *P* < 0.05), without promoting the significant GR nuclear translocation seen with DEX alone (Figure S5-2; also see Figures 1 and S2) and sustained IL-1*β* plus DEX (Figure S5-3; also see Figures 1 and S2).

We next examined whether the GR nuclear translocation or decrease in GR levels observed with IL-1*β* treatment was caused by cytokines in a nonspecific manner. We tested whether IL-18, another member of the IL-1 superfamily, had similar effects by immunofluorescence and microscopy assays. A sustained exposure to a broad range of IL-18 concentrations did not significantly modify either the DEX-driven nuclear GR translocation or cytoplasmic GR level reductions (Figure S6), indicating a degree of specificity to IL-1*β* in the effects reported here.

It is well documented in the literature that the A549 cell line expresses IL-18R*α* [64]. All the stocks of A549 cells used in the experiments were from early passages of a certified type culture collection and tested for expression of IL-18R*α*, as all reported wild type A549 cells do. Recently, Dinarello and coworkers reported the paradox that the knockdown of IL-18R*α* mRNA and protein expression in the standard type collection wild type IL-18R*α* ^+^ A549 cells results in increased IL-1*β*-induced production of inflammatory cytokines (i.e., IL-1*α*, IL-6, and IL-8) in the manipulated IL-18R*α*-deficient A549 cells, due to a dysregulation of suppressors of cytokine signaling (SOCS) [64]. Notably, this mechanism does not operate in our assay conditions because we have used wild-type IL-18R*α* ^+^ A549 cells.

Overall, therefore, prolonged exposure to IL-1*β*-alone is associated with progressive reductions in cytoplasmic GR in the absence of statistically significant GR nuclear translocation. Quantitative analyses corroborate that a sustained exposure to IL-1*β* progressively decreases the amount of cytoplasmic GR protein in a GC-unliganded manner and thereafter significantly reduces GC-liganded nuclear translocation in a cytokine-specific, IL-1*β* dose-dependent, and IL-1R1-dependent manner.

### 3.3. IL-1*β* Affects GC-Promoted GR Phosphorylation Patterns

**Figure 4:**
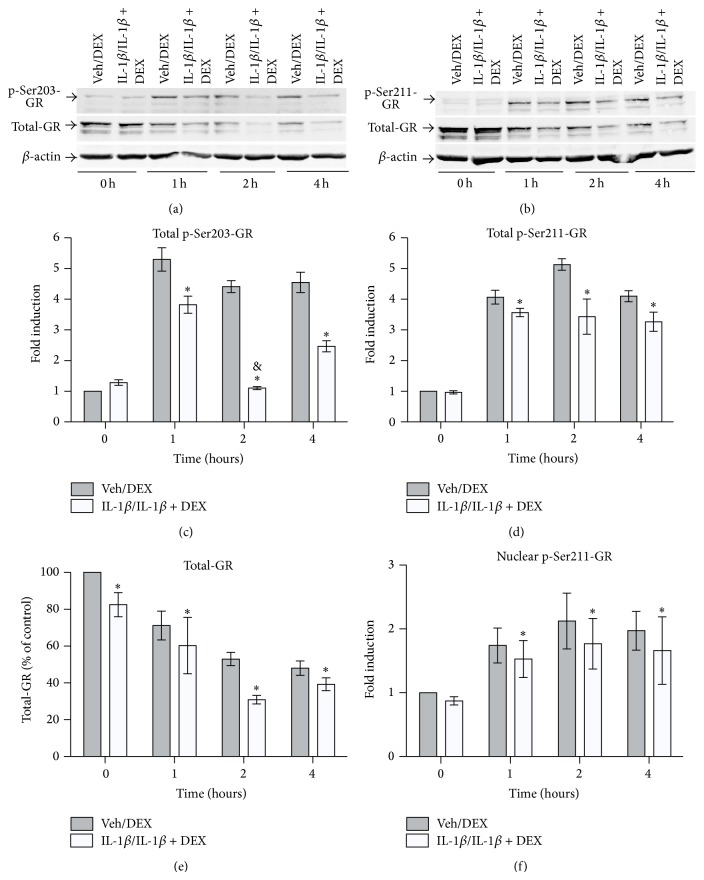
Sustained IL-1*β* conditions reduce the levels of DEX-activated cytoplasmic p-GR^Ser203^ and nuclear p-GR^Ser211^. Cells were pretreated with vehicle or 5 ng/mL IL-1*β* for 16 h followed by treatment with either 10^−8^ M DEX (veh/DEX, (gray column)) or 5 ng/mL IL-1*β* + 10^−8^ M DEX (IL-1*β*/IL-1*β* + DEX, (white column)) for 0, 1, 2, or 4 h. Whole cell lysates were prepared, normalized for total protein concentration, and tested by Western blot with p-GR^Ser203^-, p-GR^Ser211^-, and total GR-specific antibodies. Anti-*β*-actin was used to confirm that protein load was normalized in each lane before relative quantitation. Blots representative of the p-GR^Ser203^ (a) and p-GR^Ser211^ (b) analyses (*n* = 3). Quantitation of whole cell p-GR^Ser203^ (c), p-GR^Ser211^ (d), and total GR (e) protein by densitometry. Data are represented as mean ± SEM. For each condition, bars represent fold-changes in p-GR^Ser203^, p-GR^Ser211^, and GR^total^ at 1, 2, or 4 h compared to veh/DEX-treated cells at 0 h. ^∗^ *P* < 0.05, differences between sustained IL-1*β* conditions (IL-1*β*/IL-1*β* + DEX, (white column)) and DEX alone-treated cells (veh/DEX, (gray column)). ^&^ *P* < 0.05, differences in p-GR^Ser203^ induction between 2 h and 1 h post-DEX challenge under sustained IL-1*β* conditions (IL-1*β*/IL-1*β* + DEX, (white column)). (f) HCA microscopy analyses of nuclear p-GR^Ser211^ content. Results are expressed as mean ± SEM fold-induction of nuclear p-Ser^211^-GR at 1, 2, or 4 h compared to veh/DEX-treated cells at 0 h (*n* = 5). ^∗^ *P* < 0.05 IL-1*β*/IL-1*β* + DEX-treated versus veh/DEX-treated cells.

We hypothesized that the effects of IL-1*β* on GCR may involve modification of the initial steps of GR signaling (GR phosphorylation), a possibility that has not been addressed in previous studies [2, 3, 34, 35, 38–41]. GR is phosphorylated on serine residues after GC binding, which constitute codes with a profound impact on GR signaling. p-GR^Ser203^ is associated with GR nuclear translocation, and subsequent p-GR^Ser211^ activation is related to GR DNA-binding, GR association with distinct nuclear cofactors, and differential transcription of selected GR-target genes [2, 3, 34, 35, 38–41]. We initially tested whether sustained IL-1*β* conditions had an impact on GR phosphorylation codes by Western blot and densitometry studies. For standardization, blots labeled with either anti-p-GR^Ser203^ or anti-p-GR^Ser211^ were sequentially stripped and reblotted with anti-GR^total^ and anti-*β*-actin. In whole cell lysates under sustained IL-1*β* conditions, compared to DEX alone, total p-GR^Ser203^ was significantly reduced at 1, 2, and 4 h post-DEX challenge (Figures 4(a) and 4(c); ^∗^ *P* < 0.05). Notably, kinetic experiments showed an abrupt reduction in p-GR^Ser203^ protein levels at 2 hours, averaging a 75.1 ± 2.9% reduction (Figure 4(c); ^&^ *P* < 0.05 compared to sustained IL-1*β* conditions at 1 and 4 h post-DEX challenge). In stark contrast, p-GR^Ser203^ levels showed a nonstatistically significant trend towards a slow and gradual decrease at 2 and 4 h in DEX alone, consistent with previous reports [38–41, 43, 48, 65, 66]. While the kinetics of GR^total^ protein resembled the p-GR^Ser203^ curves, with diminished GR^total^ at 1, 2, and 4 h under sustained IL-1*β* conditions compared to DEX alone (Figure 4(e); ^∗^ *P* < 0.05), the reductions in p-GR^Ser203^ were twofold greater than the reductions in GR^total^ (Figures 4(c) and 4(e)). This is notable because GR^total^ protein was not significantly reduced in the cytoplasmic compartment under sustained IL-1*β* conditions compared to DEX alone (Figures 1 and S2), which suggests that sustained IL-1*β* conditions impair DEX-driven GR phosphorylation at a residue associated with efficient GR nuclear translocation, which could contribute to GCR.

Kinetic analyses of p-GR^Ser211^ activation were run in parallel to the p-GR^Ser203^ expression studies (Figure 4). As expected [2, 3, 37–39, 43, 65], after a DEX-alone challenge, the maximal p-GR^Ser211^ expression occurs at 2 h, well after the p-GR^Ser203^ peak at 1 h (Figures 4(b) and 4(d)). Under sustained IL-1*β* conditions, the 2 h p-GR^Ser211^ peak was abated, however, and there were significantly less increases in p-GR^Ser211^ at 1, 2, and 4 h post-DEX challenge compared to DEX alone (Figure 4(d); ^∗^ *P* < 0.05). The p-GR^Ser211^ activation curve was notably flattened, with levels of p-GR^Ser211^ an average 33.1 ± 5.7% lower at 2 h post-DEX challenge than DEX alone. Two results indicate that there was not a compensatory retarded p-GR^Ser211^ activation at 4 h. First, there was no significant difference in p-GR^Ser211^ activation between 2 h and 4 h post-DEX challenge under sustained IL-1*β* conditions. Second, p-GR^Ser211^ amounts were still an average 20.35 ± 2.4% lower at 4 h under sustained IL-1*β* conditions than DEX alone (Figure 4(d)). In independent experiments carried out 2 h post-DEX challenge under sustained IL-1*β* conditions, the magnitude of attenuation of whole-cell p-GR^Ser211^ activation (Figure 4(d); −33.1 ± 5.7%) and nuclear GR^total^ translocation (Figure S2B-3; −33.2 ± 4.8%) was relatively similar but less intense than the whole-cell GR^total^ reduction (−41.67 ± 5.2%), compared to DEX alone.

Considering that the transcriptional function of p-GR^Ser211^ is exerted in the nucleus but its phosphorylation is initiated in the cytoplasm [37–39, 65], we directly tested whether nuclear p-GR^Ser211^ was selectively decreased under sustained IL-1*β* conditions, compared to DEX alone. Cells were stained with the same p-GR^Ser211^-specific antibody, and nuclear p-GR^Ser211^ protein was quantified by immunofluorescence and HCA microscopy (Figure 4(f)). The amount of nuclear p-GR^Ser211^ was significantly reduced 1, 2, and 4 h after the DEX challenge under sustained IL-1*β* conditions (Figure 4(f); ^∗^ *P* < 0.05).

Together, these results indicate that preexposure to IL-1*β* reduces cytoplasmic GR^total^ levels before the DEX challenge (Figure 3) and sustained IL-1*β* conditions both attenuate DEX-driven p-GR^Ser203^ activation at the peak of ligand-driven GR nuclear translocation and reduce nuclear p-GR^Ser211^ levels (Figure 4).

### 3.4. IL-1*β* Promotes an Altered Expression Profile of Endogenous GC-Target Genes with Essential Functions in the Regulation of Inflammation and Apoptosis

The transcriptional effects of IL-1-mediated GCR have been assessed using cell lines transfected with GC-responsive reporters carrying minimal promoters, but neither GR-phosphorylation status nor endogenous GC-responsive gene expression has been studied in these systems [2, 34, 35, 37]. Recent studies on the expression of endogenous GR-driven genes have revealed unexpected mechanisms of regulation and specifically demonstrated that, in A549 cells, different GC/GR levels and GR phosphorylation changes cause selective or gene-specific effects [2, 34, 35, 41, 43, 48, 65, 66]. We therefore considered A549 cells a suitable model to ask whether sustained IL-1*β* GCR conditions differentially affect the expression of a selected set of endogenous GC/GR target genes with defined roles in inflammation and apoptosis.

**Figure 5:**
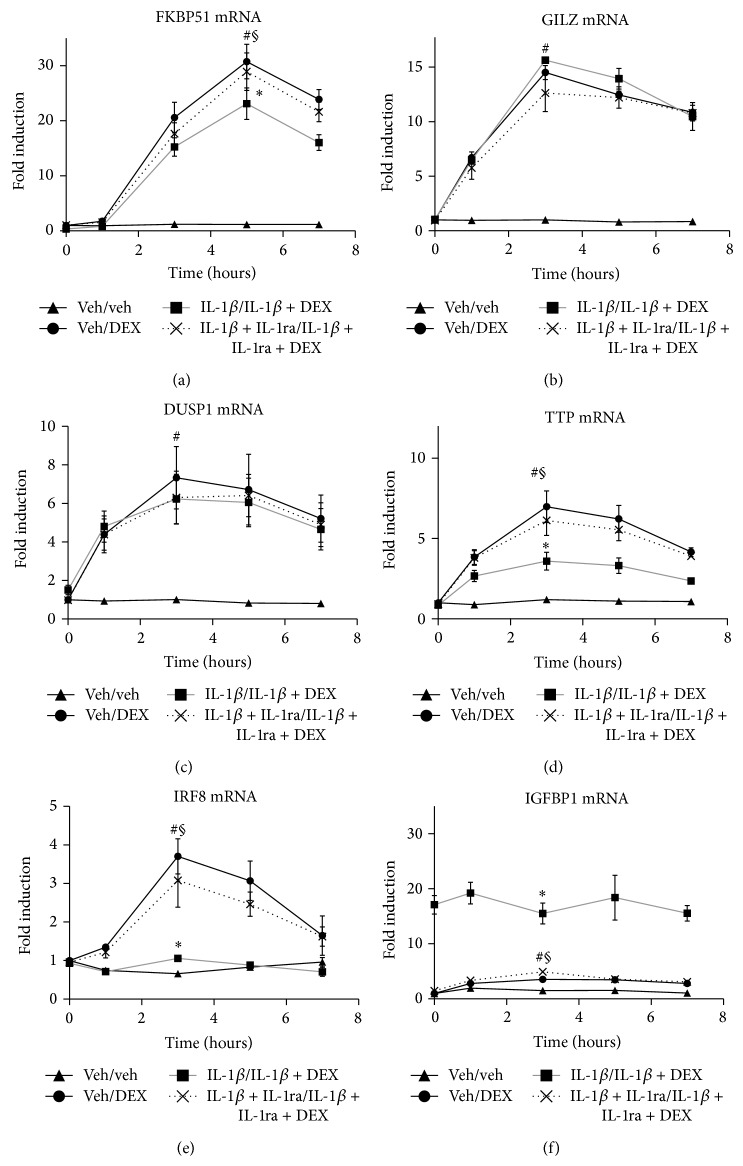
Sustained incubation with IL-1*β* promotes a split responsiveness of GC-induced anti-inflammatory genes: selective reduction in FKBP1, IRF8, and TTP, superinduction of IGFBP1 and unchanged DUSP1 and GILZ mRNA expression. Cells were cultured in complete medium (veh/veh, ▲, solid line), pretreated 16 h in vehicle, then treated with 10^−8^ M DEX alone for the indicated times (veh/DEX, ●, solid line), subjected to sustained IL-1*β* conditions (16 h 5 ng/mL IL-1*β*/5 ng/mL IL-1*β* + 10^−8^ M DEX for the indicated times, ■, solid grey line), or treated under sustained IL-1*β* conditions in the presence of IL-1ra (16 h 5 ng/mL IL-1*β* + IL-1ra/5 ng/mL IL-1*β* + IL-1ra + 10^−8^ M DEX for the indicated times, ×, dashed line). After the indicated treatments, the cells were collected and mRNA extracted and quantitated by real-time RT-PCR. The time-course curves represent the mean ± SEM fold-changes in the expression of FKBP51 (a), GILZ (b), DUSP1 (c), TTP (d), IRF8 (e), and IGFBP1 (f) mRNAs, for each culture condition compared to veh/veh-treated cells at 0 h (*n* = 5). ^#^ *P* < 0.05, veh/DEX-treated versus veh/veh-treated cells. ^∗^ *P* < 0.05, sustained IL-1*β* conditions versus DEX alone-treated cells. ^§^ *P* < 0.05, differences between cells under sustained IL-1*β* conditions in the presence or absence of IL-1ra.

We investigated by real-time RT-PCR kinetic assays whether sustained IL-1*β* conditions, compared to DEX or IL-1*β*-alone, reprogram the mRNA expression of six GR-driven genes (FKBP51, GILZ, DUSP1, TTP, IRF8, and IGFBP1; Figure 5). Among the six genes studied, FKBP51 was the most highly induced mRNA after treatment with DEX alone (29.8 ± 3.6-fold increase in 10^−8^ M DEX compared to vehicle, Figure 5(a); ^#^ *P* < 0.05), as expected [37, 39, 40, 43, 65]. FKBP51 expression is a biomarker of GC-responsiveness that correlates with GC availability and nuclear GR amounts* in vivo* and* in vitro* [1, 2, 5, 37, 39, 65]. Under sustained IL-1*β* conditions, the peak of FKBP51 at 5 h was significantly lower than with DEX alone (Figure 5(a); ^∗^ *P* < 0.05), with FKBP51 reduced by an average of 29.61 ± 3.4%. Significant FKBP51 reductions occurred after GC challenges at a broad range of DEX concentrations under sustained IL-1*β* conditions compared to DEX alone (Figure S7A; 10^−9^ M DEX, ^∗∗^ *P* < 0.005; 10^−8^ M DEX, ^∗^ *P* < 0.05; 10^−7^ M DEX, ^∗^ *P* < 0.05; average reductions 59 ± 2%, 30 ± 3%, and 24 ± 1%, resp.). Notably, IL-1*β* alone did not significantly modify FKBP51 expression, which was similar to vehicle at all time points (Figure S8A). This is consistent with FKBP51 as biomarker of GC-responsiveness that is not induced by proinflammatory agents alone [2, 33, 37]. Overall, sustained IL-1*β* conditions significantly reduced GC-responsiveness at a broad range of DEX concentrations as assessed by standard FKBP51 assays. The magnitudes of the FKBP51 reductions were remarkably similar to the reductions in nuclear GR translocation (Figures 1 and S2), consistent with the proposal that IL-1R1 can promote GCR in different conditions [1, 2, 5, 33–35].

It is noteworthy that the 10^−11^ to 10^−5^ M DEX dose range is the uniformly used one in GR signaling studies and would span well below and above the expected plasma levels in patients receiving the DEX doses recommended for anti-inflammatory treatment by European government agencies (http://www.medicines.org.uk/emc/ingredient/328/dexamethasone).

The remaining genes are shown in decreasing order of GC responsiveness (Figures 5(b)–5(f), Figure S8B-F, all significant, ^#^ *P* < 0.05). Surprisingly, the GC-responsiveness of individual genes shows distinct changes under sustained IL-1*β* conditions (Figure 5). The modified responses fall into a maximum of 5 possible categories. First, DUSP1 did not display significant GCR. Second, GILZ only showed significant inhibition at low DEX under sustained IL-1*β* conditions. Thus, after a DEX challenge under sustained IL-1*β* conditions, GILZ (Figure 5(b)) and DUSP-1 (Figure 5(c)) mRNA levels were not significantly different than those with DEX alone over a broad range of DEX concentrations, except GILZ at 10^−9^ M DEX (Figure S7B, ^∗^ *P* < 0.05). This was expected because GILZ has significant p-GR^Ser211^ dependence only at low doses of GC (e.g., 10^−9^ M) [40, 48, 49], and p-GR^Ser211^ nuclear levels are reduced in this cell model (Figures 4(d) and 4(f)).

Third, TTP expression showed a significantly blunted induction after a DEX challenge under sustained IL-1*β* conditions (Figure 5(d); ^∗^ *P* < 0.005, with an average peak level reduction of 63.16 ± 3.9% compared to DEX alone). Compared to DEX alone, significant TTP reductions occurred with a broad range of DEX concentrations under sustained IL-1*β* conditions (Figure S7D; 10^−8^ M DEX, ^∗∗^ *P* < 0.005; 10^−7^ M DEX, ^∗^ *P* < 0.05; 10^−6^ M DEX, and ^∗^ *P* < 0.05; average reductions 63.16 ± 3.75%, 47.1 ± 1.75%, and 61.54 ± 3.26%, resp.). Thus, FKBP51 and TTP represent two cases of different extents of “partial” GCR: TTP had an average relative reduction of ~2/3 (Figure 5(d)), greater than the 1/3 decrease observed in FKBP51 (Figure 5(a)). Contrary to the case of GILZ, both FKBP51 and TTP GCR occur at high GC doses, a feature of GCR relevant in clinical practice [1, 2, 5, 47].

Fourth, the induction of IRF8 mRNA expression was abrogated after a DEX challenge under sustained IL-1*β* conditions (Figure 5(e); ^∗^ *P* < 0.05, with an average peak level reduction of 100.89 ± 5.25% compared to DEX alone). The loss of IRF8 induction under sustained IL-1*β* conditions occurred at all DEX concentrations that alone caused induction (Figure S7E; ^∗^ *P* < 0.05). Therefore, there was an IRF8 knockdown or “complete” GCR for IRF8 responsiveness.

Fifth, IGFBP1 mRNA expression was super-induced after a DEX challenge under sustained IL-1*β* conditions (Figure 5(f); ^∗^ *P* < 0.05, with an average super-induction of 514.29 ± 27.82% compared to DEX alone). The superinduction occurred after the GC challenge under sustained IL-1*β* conditions with all DEX concentrations that alone caused induction (Figure S7F; ^∗∗^ *P* < 0.005). These results are not unexpected because the preincubation with IL-1*β* was a much more potent stimulus for IGFBP1 induction than DEX alone (Figure S8F). Finally, IL-1ra reverted reprogramming of the expression of FKBP51, TTP, IRF8, and IGFBP1 genes from the sustained IL-1*β* condition profiles to patterns not significantly different from those observed after the DEX-alone challenges (Figures 5(a), 5(d), 5(e), and 5(f); ^§^ *P* < 0.05). This indicates that these altered expression profiles with sustained IL-1*β* conditions are dependent on IL-1R1.

Overall, the distinct patterns of responsiveness shown by individual genes demonstrate an unexpected degree of specificity that reveals that sustained IL-1*β* conditions cause a split-resistance to the transactivation of prominent anti-inflammatory genes by glucocorticoids, which can be reverted by IL-1ra.

Recent GCR models propose that sustained cytokine exposure generally compromises GC transactivation potential of endogenous GR-driven genes, with global loss of prominent anti-inflammatory functions [2, 33, 47, 49–53]. Here we show that sustained IL-1R1 signaling alone reduces the level of cytoplasmic GR in a time-dependent and IL-1*β* dose-dependent manner that is GC ligand- and GR nuclear translocation-independent. DEX treatment under sustained IL-1*β* conditions further reduces the cytoplasmic GR level, which is accompanied by a significant decrease in GR nuclear translocation. Six endogenous GR-driven anti-inflammatory genes have individual GC-responses: some show complete or partial GCR whereas others have unchanged or increased gene expression under sustained IL-1*β* conditions. This supports a IL1R1-driven “split GCR” model, distinct from current “global GCR” models [1, 2, 5, 33–35, 52, 53].

**Figure 6:**
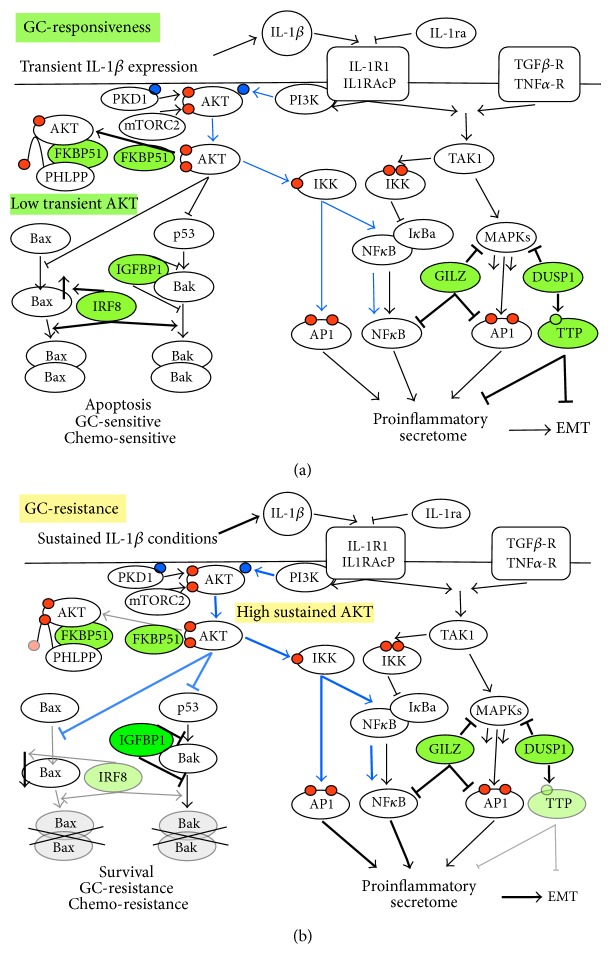
IL-1R1 signal transduction and negative regulation of GR-induced genes with known functions in inflammation and apoptosis: evidence of split GCR under sustained IL-1*β* conditions and proposed association with chemo- and GC-resistance phenotypes and EMT. (a) Signaling through the TAK1 and AKT parallel transduction nodes under normal IL-1R1 signaling conditions [44]. In the right node, TAK1 activates the three MAPK signaling cascades, including the JNK-AP1 pathway and the classical NF*κ*B pathway. In the left node, AKT transiently promotes survival by inhibiting p53 and Bak/Bax-mediated apoptosis (thin black inhibition arrows) and also triggers a separate NF-*κ*B/AP1 activation pathway (thin blue arrows) [47]. The schematic superimposes the most prominent effects mediated by the 6 selected GC-inducible genes (FKBP51, GILZ, DUSP1, TTP, IRF8, and IGFBP1, highlighted in green) in the regulation of IL-1R1 signaling under noninflammatory conditions. The level of wild-type FKBP51 contributes to low transient AKT activity, while IRF8 increases Bax expression and Bax/Bak pathway activities (bold arrows) which counteracts the AKT and IGFBP1 upstream survival regulation. GILZ, DUSP1, and TTP exert their regulatory functions as indicated by the bold inhibition arrows [2, 47–56]. (b) Under sustained IL-1*β* conditions, GC-driven transcription of FKBP51 is significantly reduced, which allows sustained AKT signaling (bold blue lines), which in turn has been associated with apoptosis- and chemo- and GC-resistance [52, 57]. IRF8 knockdown (in faded green) would reinforce the antiapoptotic AKT effects due to lower Bax expression and weak inhibition of other activities in the same pathway that lead to Bax-Bax pore formation (faded gray arrows) [55, 56]. The IGFBP1 overexpression would reinforce the antiapoptotic effects of AKT by further inhibiting activities that lead to Bak-Bak pore oligomerization [58] in the alternative pathway (faded and crossed-out). While GILZ and DUSP1 expression is unaffected in the IL-1*β*-induced split GCR model, sustained AKT activation might still reinforce the expression of the proinflammatory secretome via the noncanonical NF-*κ*B/AP1 pathway (bold blue arrows) [47]. The latter might be potentiated by the reduction in TTP expression (2/3 less), a knockdown that has been also associated with EMT [54, 59].

Recent research has focused on the most prominent genes that restrain IL1R1-driven signaling by interdependent strata of negative regulatory pathways and has mapped the role of essential GR-driven genes that control IL-1*β*-mediated inflammation under GC-responsive conditions [31, 48, 49]. A schematic of IL-1R1 signaling depicts two major transduction nodes (Figure 6(a)) [22, 30, 47–50]. The first node includes TAK1 (transforming growth factor activated kinase 1, shared by TGF-R, TNF-R, and IL-1R1) which activates several MAPK signaling cascades, such as the JNK-AP1 pathway and the canonical NF*κ*B pathway. In the second node, phosphatidylinositol-3-kinase (PI3K) and AKT promote survival by inhibiting p53 and Bak/Bax-mediated apoptosis and trigger separate NF-*κ*B/AP1 activation pathways (Figure 6(a), blue arrows) [4, 23–26, 29, 47, 49–54]. The most prominent effects mediated by the six selected GC-inducible genes in the regulation of IL-1R1 signaling by multitiered feedback loops (highlighted in green) are also summarized in Figure 6(a). First, FKBP51 is a scaffold protein in the AKT and TAK1 signal nodes and hence regulates the downstream scaffold-associated survival/apoptosis pathways [28, 52, 53, 55, 56, 67]. Second, GILZ directly binds to and inhibits NF-*κ*B as well as MAPKs and their downstream nuclear factors, such as JNK/AP1 [31, 48, 58]. Third, DUSP1 upregulates TTP activity without modifying the total levels of TTP protein by dephosphorylation of p-TTP and is a pivotal negative feedback regulator of MAPK signaling. It is therefore important in inflammatory cytokine secretion and resistance to MAPK-driven apoptosis [22, 30, 48–50, 52]. Fourth, TTP degrades many different mRNA targets known to promote inflammation, angiogenesis, and metastasis (the proinflammatory secretome) and also represses the epithelial-mesenchymal transition (EMT) [4, 23–26, 29, 54, 57]. Fifth, IRF8 is an IFN- and GC-induced gene whose expression is inversely correlated with inflammation and apoptosis-resistance. IRF8 increases apoptosis due to increased expression of several molecules in the death signaling pathways that permeate mitochondrial and endoplasmic reticulum membranes by favoring the assembly of proapoptotic Bax/Bak protein pore-opening complexes [28, 52, 55–57, 67]. Lastly, sixth, while IRF8 increases Bax expression, IGFBP1 is a direct binding partner of Bak, a mechanism by which it prevents the initiation of the death program by inhibiting Bak oligomerization and hence the opening of Bak/Bak pores [58, 67].

Figure 6(b) summarizes how the genes' functions in IL-1R1 signal regulation and their combined expression changes in the split GCR model might represent complementary losses of function in the AKT node. Specifically, reduced FKBP51 expression results in Ser^473^-AKT hyperphosphorylation in A549 cells [52, 58], which increases AKT signal strength and duration, shown as bold blue lines. Sustained AKT activity is a negative regulator of GR nuclear translocation, GC-target gene transactivation, and apoptosis [28, 55–58, 67]. These combined AKT activities contribute to chemo- and GC-resistance by mechanisms that limit Bak/Bax protein oligomerization in the pores that trigger the lethal hit [49, 52, 55–58, 67]. Simultaneous loss of Bak + Bax function render cells resistant to apoptosis via all Bcl-2 family signal pathways [6–8, 67], summarized by the faded and crossed-out Bax/Bax and Bak/Bak pores in Figure 6(b). It is known that increased IGFBP1 prevents Bak-mediated death by inhibiting Bak oligomerization [4, 6–8, 50–52, 58]. Further, IRF8 epigenetic knockdown results in apoptosis resistance by loss of function of more than one molecule in the two activation pathways and strongly downregulates Bax, the Bak functional complementation gene [23–26, 28, 48, 49, 54–56, 58, 59, 67]. Hence, a FKBP51 decrease, IGFBP1 increase, and IRF8 knockdown are a reinforced combination of elements known to separately provide cell survival under potent stress, inflammation, or genotoxic conditions, and it has been reported that these individual gene expression changes promote IFN-, chemo-, and GC-resistance [4, 46, 49, 52, 55–58, 67, 68]. Together, this suggests that IL-1*β*-driven modification of the functions of these three genes may be the basis of an unexpected multiple drug resistance mechanism, involving drugs with very different mechanisms of action and which are essential to treat prevalent and life-threatening cancer and inflammatory diseases.

A549 lung cancer epithelial cells were chosen for this study because they have been instrumental to defining GR signaling mechanisms and the complexity of the regulatory activities of endogenous GR-transactivated genes. Herein, their reprogramming to a split GCR occurs after 18 h under sustained IL-1*β* conditions. It has been proposed that the senescence associated secretory phenotype (SASP) exploits chronic IL-1R1 signaling and the IL-1*β* paracrine proinflammatory secretome to cause concurrent inflammation, degenerative syndromes, and cancer from middle age onwards [6–8, 47]. SASP also blocks p53-driven apoptosis and promotes GCR but is further associated with epithelial-mesenchymal transition (EMT) [4, 6–8, 47, 50–52]. In this regard, it is known that IL-1*β* promotes EMT in tumors and healthy tissues, such as placental trophoblasts, in counterbalance with GC, and that TTP knockdown causes EMT [23–26, 33, 48, 49, 54, 59]. More related to our* in vitro* model, there have been reported TTP knockdown phenotypes and EMT phenotypes in A549 cells and cancer tissues* in vivo* after extended IL-1*β* exposure [4, 33, 46, 63, 68]. Here, we observed a significant two-thirds reduction in TTP expression (Figures 5(d) and 6(b)). Interestingly, experiments aiming to completely knock down TTP protein expression by increasing the sustained IL-1*β* time up to 72 h resulted in cells with a morphological phenotype (Figure S9), which did not occur in 18 h short-term cultures (Figures 1–5). In fact, it is reminiscent of the fully characterized EMT morphology already reported in A549 cells treated long-term with IL-1*β* [46]. The characterization of the effects of long-term exposure to IL-1*β* alone and EMT mechanisms was not an aim of our project. Further experiments will be required to characterize the several discussed potential mechanisms of action and their biological implications (Figure 6) in different models.

Previous work helps suggest potential split GCR mechanisms in SASP that may be reverted by IL-1ra. Campisi and coworkers recently reported that GCs only suppress the secretion of “selected” SASP components and not others such as IGFBP1 [9, 33, 48, 51, 69]. This is similar to the selective GC gene responsiveness in our split GCR: the “selected suppression” required that GC was present during the whole extended period of SASP establishment (1 week) and added IL-1*α* promoted the SASP rescue under the sustained GC conditions. The authors monitored the SASP rescue by the IL-6 secretion recovery and attributed the SASP rescue to increased IL-1R1-driven NF*κ*B signals [33, 51]. Importantly, Cahill and Rodgers identified novel AKT-dependent pathways from IL-1R1 to IL-6 transcriptional activation [33, 47], depicted as blue arrows in Figure 6(a). One leads to AP1-dependent IL-6 induction via direct phosphorylation of IKK*α*-Thr^23^ by AKT; the other connects AKT and IKK*α*-Thr^23^ to the NF*κ*B activation pathway downstream from the TAK1 node, which canonically activates MAPK/AP1, NF*κ*B, and IL-6 [23–26, 47–49]. The bold blue arrows in Figure 6(b) illustrate how sustained AKT activity would promote high AP1/NF*κ*B activities bypassing the TAK1 node. The TAK1 node is shared by distinct cytokine receptors, like TNF-R (Figure 6). Notably, TNF*α* injections did downregulate the level of GR protein* in vivo* [4, 6–10, 12, 14, 16, 19–21, 33, 49–57], as shown here for IL-1*β in vitro*. GR level has been proposed to determine the potency of subsequent anti-inflammatory GR-driven responses, as shown in gene-dosage studies [33, 47, 63]. GILZ and DUSP1 expression is essential for mice to survive MAPK-driven apoptosis and hence TNF*α* or IL-1*β*-induced shock [2, 9, 33, 47–56, 69]. Here, their expression is notably unchanged under sustained IL-1*β* conditions (Figure 6(b)). Lethal TNF*α* doses, however, knock down GILZ expression [33], leading Libert and coworkers to propose that GR reductions generally compromise GC transactivation potential and that TNF*α* amplifies its own proinflammatory potential by inducing a “global” GCR that blocks the GILZ, DUSP1, and TTP endogenous GR-mediated brakes on inflammation [33]. Our results, obtained under sustained IL-1*β* conditions, lead us to propose a not-mutually-exclusive split GCR model, operating under different cytokine conditions. These might combine cell survival (driven by FKBP51, IRF8, and IGFBP1 clustered changes) and inflammation (as the TTP knockdown amplifies the proinflammatory potential of the IL-1*β*-induced paracrine secretome [23–26, 48, 49]).

Concern about the involvement of IL-1*β* in the pathogenesis of prevalent diseases is rapidly growing, especially in view of its role in lethal diseases and in the paracrine propagation of SASP-associated cancer and chronic degenerative syndromes, which frequently associate with chemotherapy- and/or GC-resistance [4, 6–10, 12, 14, 16, 19–21, 49–57]. The finding of a split GCR reversal by IL-1ra in the* in vitro* model will merit further investigation in preclinical and clinical assay conditions to assess whether the amplification of the IL-1*β* proinflammatory cascade potential can be disrupted in these conditions.

## 4. Conclusions

Exposure of cells to sustained IL-1*β* conditions has a profound impact on subsequent response to GC: cytoplasmic GR level, GR^Ser203^ and GR^Ser211^ phosphorylation, and GR nuclear translocation are all reduced. Although current GCR models propose that sustained cytokine exposure generally compromises GC transactivation, we show here that sustained IL-1*β* exposure promotes a “split GCR” model (reduced GC-induced FKBP51, TTP, and IRF8 mRNAs are accompanied by an increased expression of GC-induced IGFBP1, and no changes in GC-induced DUSP1 and GILZ). Thus, sustained IL-1*β* conditions can lead to a selective modulation of GC-induced gene transcription, known to cause resistance to apoptosis and chemotherapy in addition to GCR, rather than causing a general GC-induced gene shutdown. Notably, the integrated gain and loss of gene functions reported here in the “split GCR” model are reverted by IL-1R1 antagonist. Together, it provides an alternative explanation for the chemo- and GC-resistance associated to prevalent diseases with elevated IL-1*β* and provides rational design novel therapeutical strategies.

## Supplementary Material

The Supplementary Material contains nine supplementary figures that provide access to essential data that do not appear in the main article but that are complementary to the experiments reported in the final paper and required to reach all the final conclusions. These data are peer-reviewed and are subject to the same criteria as data in the main article.
