# Supplementary material for: Sustained Interleukin-1β Exposure Modulates Multiple Steps in Glucocorticoid Receptor Signaling, Promoting Split-Resistance to the Transactivation of Prominent Anti-Inflammatory Genes by Glucocorticoids
Source: Mediators Inflamm. 2015 Apr 22;2015:347965. doi: 10.1155/2015/347965 (PMC4421076; doi:10.1155/2015/347965)
Supplement: Supplementary file 1 — The Supplementary Material contains nine supplementary figures that provide access to essential data that do not appear in the main article but that are complementary to the experiments reported in the final paper and required to reach all the final conclusions. These data are peer-reviewed and are subject to the same criteria as data in the main article. [file 347965.f1.pdf]

Supplementary Material

Sustained interleukin-1β exposure modulates multiple steps in glucocorticoid receptor signaling, promoting split-resistance to the transactivation of prominent anti-inflammatory genes by glucocorticoids

Pedro Escoll, Ismael Ranz, Norman Muñoz-Antón, Ana van-den-Rym, Melchor Alvarez-Mon,

Carlos Martínez-Alonso, Eva Sanz and Antonio de-la-Hera

The Supplementary Material below contains nine supplementary figures that provide access to essential data that do not appear in the main article but that are complementary to the experiments reported in the final version of the paper online and required to reach all the final conclusions. These data are peer-reviewed and are subject to the same criteria as data in the main article. Each of the nine figures is displayed in a separate page with their title and legends placed below each corresponding figure.

Index for the Supplementary Figures:

page 2: **Figure S1. HCA analysis strategy.**

page 3: **Figure S2. Sustained IL-1β conditions reduce DEX-driven nuclear GR protein levels without a reciprocal increase in cytoplasmic GR levels.**

page 4: **Figure S3. Sustained IL-1β conditions do not significantly modify the DEX-induced downregulation of GR-α or GR-β mRNA.**

page 5: **Figure S4. The reduction in whole-cell GR protein level promoted by sustained IL-1β conditions is reverted by IL-1ra**

page 6: **Figure S5. IL-1β alone does not promote significant nuclear GR translocation.**

page 7: **Figure S6. Sustained IL-18 conditions do not reproduce the effects promoted by sustained IL-1β conditions.**

page 8: **Figure S7. GC dose-response curves of FKBP51, GILZ, DUSP1, TTP, IRF8, and IGFBP1 mRNA expression under DEX alone or sustained IL-1β conditions.**

page 9: **Figure S8. Kinetics of FKBP51, GILZ, DUSP1, TTP, IRF8, and IGFBP1 mRNA expression after IL-1β-or DEX-alone stimulation.**

page 10: **Figure S9. Prolonged 72h IL-1β exposure promotes changes in the morphological phenotype of A549 cells reminiscent of epithelial-mesenchymal transition (EMT).**

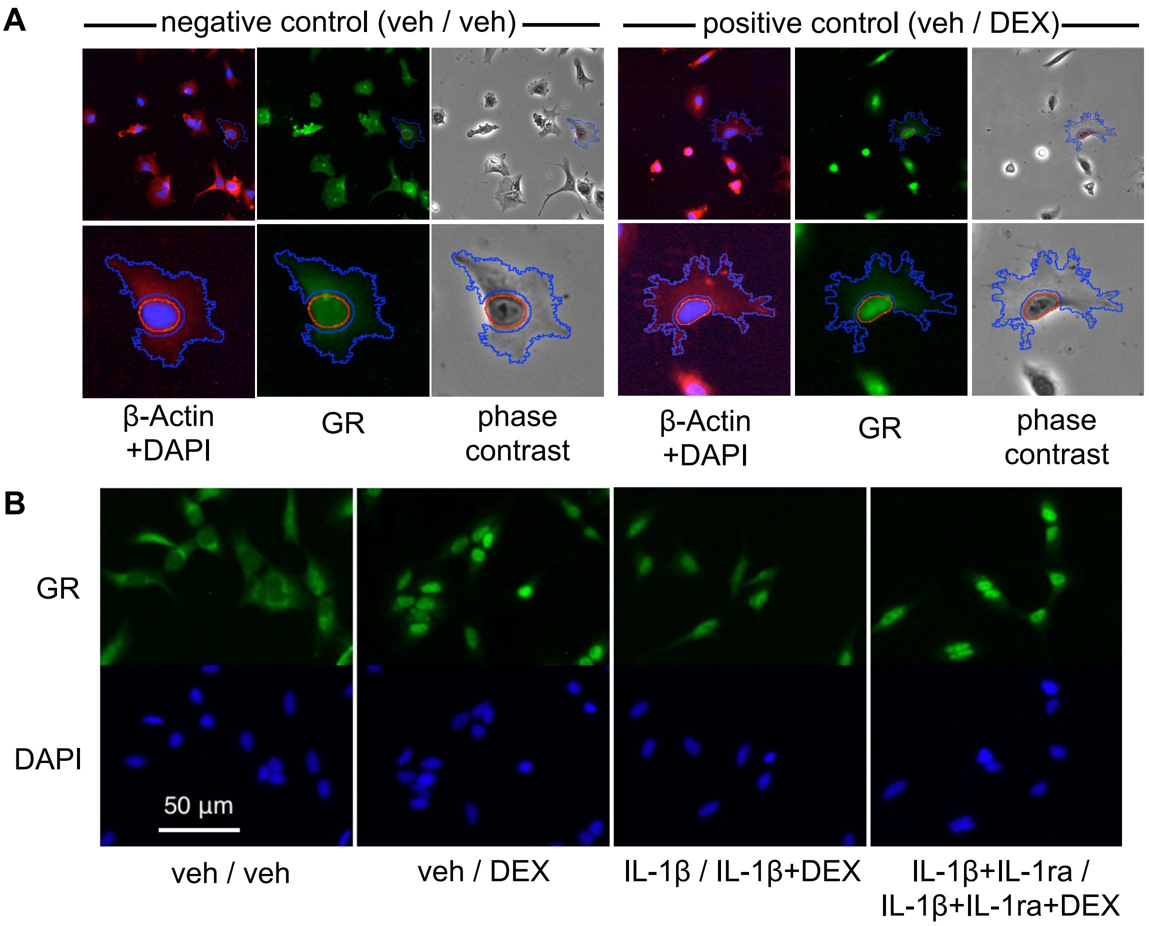

Figure S1. HCA analysis strategy. Cells were pretreated with either vehicle (veh) or 5ng/ml IL-1β for 16h, and then treated for 1h with either veh, 10^{-8} M DEX or 5ng/ml IL-1β+10^{-8} M DEX, in the absence or presence of 1μg/ml IL-1ra, as indicated in the Figure. After culture, cells were fixed, permeabilized, and stained with β-actin- and GR-specific antibodies. They were then labeled with secondary fluorescent antibodies and DAPI, mounted, and subjected to automated High Content Analyses (HCA) three-color fluorescence microscopy. β-actin and DAPI staining were used to distinguish the cytoplasmic and nuclear regions of interest (ROI), respectively, in more than 2500 cells per condition. (**A**) ROI segmentation criteria in GR-translocation negative (veh/veh, left) and positive (veh/DEX, right) controls. β-Actin (red) + DAPI (blue), GR (green) and phase contrast (gray) images of representative fields are shown. The outlines drawn around the nucleus and cytoplasm are examples of the automated segmentation performed by the HCA imaging system in individual cells. (**B**) Representative fields of GR (green) and DAPI (blue) staining in each condition: veh/veh, veh/DEX, sustained IL-1β conditions (IL-1β/IL-1β+DEX) and sustained IL-1β conditions plus IL-1ra (IL-1β+IL1-ra/IL-1β+IL1-ra+DEX).

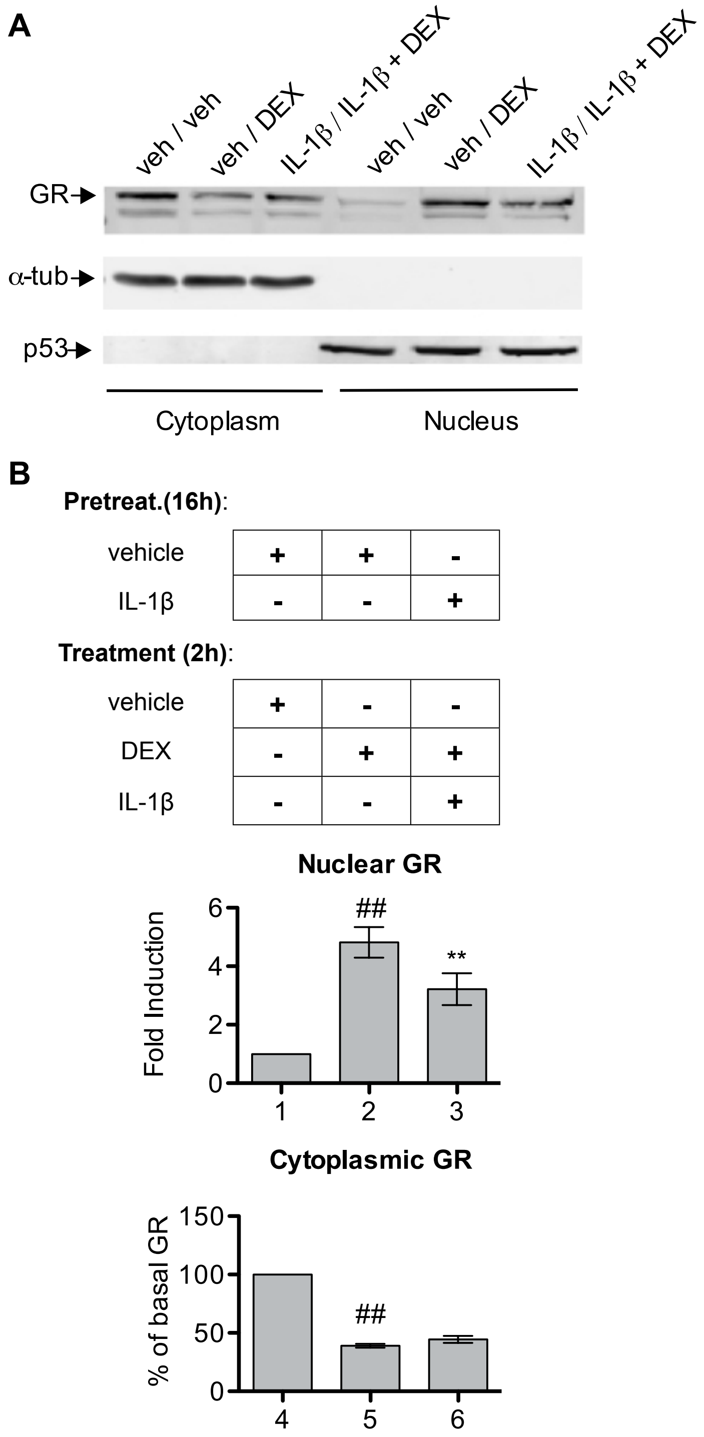

Figure S2. Sustained IL-1β conditions reduce DEX-driven nuclear GR protein levels without a reciprocal increase in cytoplasmic GR levels. Cells were pretreated for 16h with vehicle or 5ng/ml IL-1β, and then treated for 2h with either vehicle (B1 and B4, veh/veh) or stimulated with either 10^{-8} M DEX alone (B2 and B5, veh/DEX) or 5 ng/ml IL-1β+10^{-8} M DEX (B3 and B6, IL-1β / IL-1β+DEX), as shown in the table. **A**) The nuclear and cytoplasmic compartments were then fractionated and their lysates studied by Western blot with anti-GR antibody (n=10). Anti-α-tubulin (α-tub) and -p53 antibodies are controls for correct cytoplasmic and nuclear compartment fractionation, respectively. **B)** Nuclear and cytoplasmic GR protein quantification by densitometry. Data are shown as mean±SEM fold-changes in the nuclear and cytoplasmic GR under the indicated conditions compared to cells in vehicle. $^{##}$p<0.005, differences in either nuclear or cytoplasmic GR protein levels between veh/DEX-treated (B2, B5) and veh/veh-treated cells (B1, B4). **p<0.005, difference in nuclear GR between IL-1β/IL-1β+DEX treated (B3) and veh/DEX-treated cells (B2).

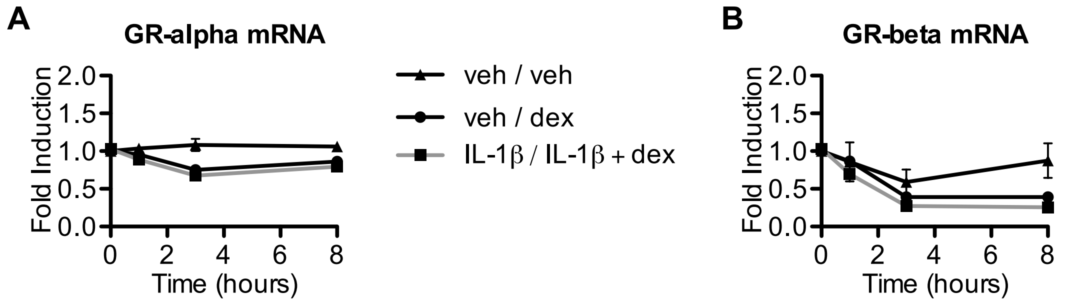

Figure S3. Sustained IL-1β conditions do not significantly modify the DEX-induced downregulation of GR-α or GR-β mRNA. Cells were pretreated with 5ng/ml IL-1β or vehicle for 16h and were then treated for 1, 3 or 8h with vehicle (veh/veh, ▲, solid line), 10^{-8} M DEX (veh/DEX, ●, solid line) or sustained IL-1β conditions (5ng/ml IL-1β/5ng/ml IL-1β+10^{-8} M DEX, ■, solid grey line). GR-α (**A**) and GR-β (**B**) mRNA expression levels were analyzed by real-time quantitative RT-PCR. The time-course curves represent the mean±SEM fold-changes in GR-α and GR-β mRNA for each culture condition compared to veh/veh-treated cells at 0h (n = 4).

A

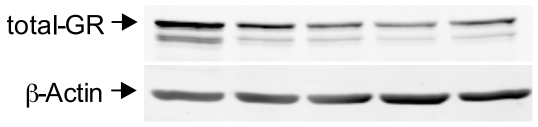

Pretreat.(16h):

vehicle	+	+	+	-	-
IL-1β	-	-	-	+	+
IL-1ra	-	-	-	-	+

Treatment (2h):

vehicle	+	-	-	-	-
DEX	-	+	+	+	+
IL-1β	-	-	+	+	+
IL-1ra	-	-	-	-	+

B

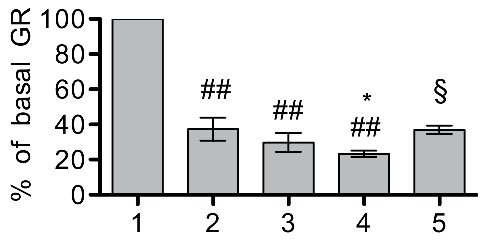

Figure S4. The reduction in whole-cell GR protein level promoted by sustained IL-1β conditions is reverted by IL-1ra. Cells were pretreated for 16h with either vehicle or 5ng/ml IL-1β in the presence or absence of IL-1ra, and then treated for 2h with vehicle (B1, veh/veh), 10^{-8} M DEX alone (B2, veh/DEX), simultaneous addition of 5 ng/ml IL-1β and 10^{-8} M DEX (B3, veh/IL-1β+DEX), sustained IL-1β conditions (B4, IL-1β/IL-1β+DEX) or sustained IL-1β conditions plus IL-1ra (B5, IL-1β+IL-1ra/IL-1β+IL-1ra+DEX), as indicated in the table. Whole cell lysates were prepared and analyzed by Western blot with anti-GR and -β-actin antibodies. (**A**) Blots of a representative experiment (n=3). (**B**) Quantitation of whole-cell GR protein by densitometry. Data are represented as mean±SEM percentage of total GR protein amount compared to veh/veh treatment. $^{\#\#}p<0.005$, difference in GR protein levels between cells treated with veh/DEX (B2), veh/IL-1β+DEX (B3) or IL-1β/IL-1β+DEX (B4) versus veh/veh-treated cells (B1). *$p<0.05$ IL-1β/IL-1β+DEX-treated (B4) versus veh/DEX-treated cells (B2). $^{\S}p<0.05$ IL-1β+IL-1ra/IL-1β+IL-1ra+DEX-treated (B5) versus IL-1β/IL-1β+DEX-treated cells (B4).

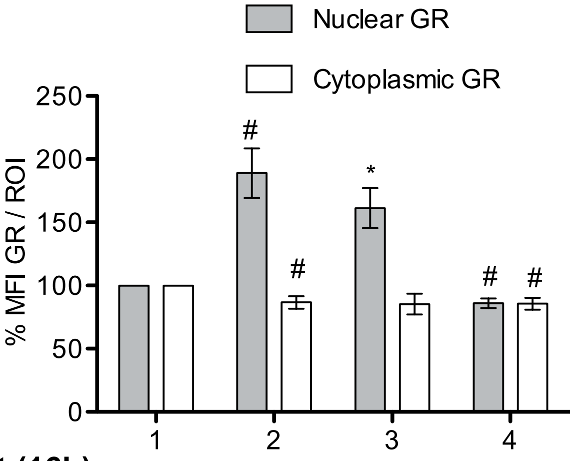

Pretreat.(16h):

vehicle	+	+	-	-
IL-1β	-	-	+	+

Treatment (1h):

vehicle	+	-	-	-
DEX	-	+	+	-
IL-1β	-	-	+	+

Figure S5. IL-1β alone does not promote significant nuclear GR translocation. Cells were pretreated with either vehicle or 5ng/ml IL-1β for 16h, and then cultured for 1h in medium without stimulus, or stimulated with either 10^{-8} M DEX, 5ng/ml IL-1β, or 5ng/ml IL-1β+10^{-8} M DEX, as indicated in the table. The cells were processed for HCA microscopy analysis as detailed in Figure S1. Nuclear (■) and cytoplasmic (□) GR intensity results are expressed as % Mean Fluorescence Intensity (MFI)/nuclear or cytoplasmic ROI±SD (n = 3), relative to GR content in veh/veh-treated cells. $^{\#}p<0.05$ veh/DEX-treated (2) and IL-1β/IL-1β-treated (4) versus veh/veh-treated cells (1). $^{*}p<0.05$ IL-1β/IL-1β+DEX-treated (3) versus veh/DEX-treated cells (2).

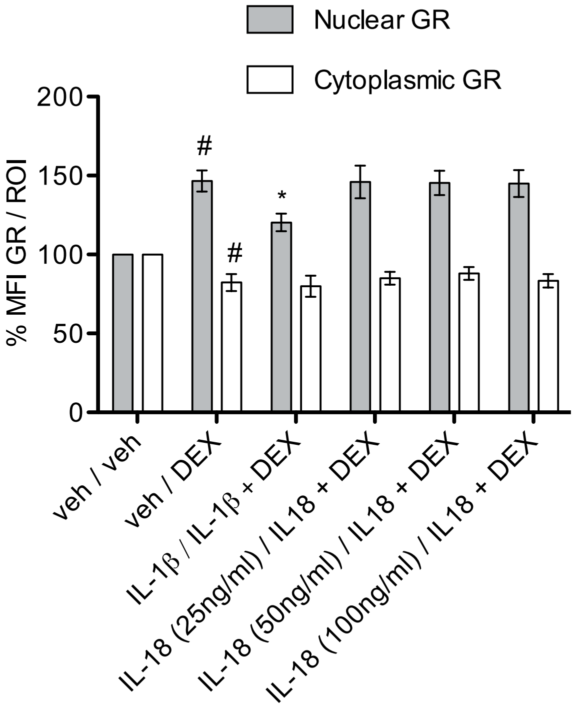

Figure S6. Sustained IL-18 conditions do not reproduce the effects promoted by sustained IL-1β conditions. Cells were pretreated with vehicle, 5ng/ml IL-1β, or 25, 50 or 100ng/ml IL-18 for 16h, and then treated for 1h with either vehicle, 10^{-8} M DEX, 5ng/ml IL-1β+10^{-8} M DEX or 25, 50 or 100ng/ml IL-18+10^{-8} M DEX, as indicated. The cells were fixed, permeabilized, and stained with anti-β-actin and anti-GR antibodies. The slides were then stained with DAPI, mounted, and subjected to fluorescence microscopy imaging. Acquired images were analyzed by High Content Analysis (HCA) as in Figure S1. Each histogram bar represents HCA results and includes 25 microscope fields and at least 2,500 cells. Nuclear (■) and cytoplasmic (□) GR intensity results are expressed as % Mean Fluorescence Intensity (MFI)/nuclear or cytoplasmic ROI±SD (n = 3), relative to GR content in veh/veh-treated cells. $^{\#}p<0.05$ veh/DEX-treated versus veh/veh-treated cells. *p<0.05 IL-1β/IL-1β+DEX-treated versus DEX-treated cells.

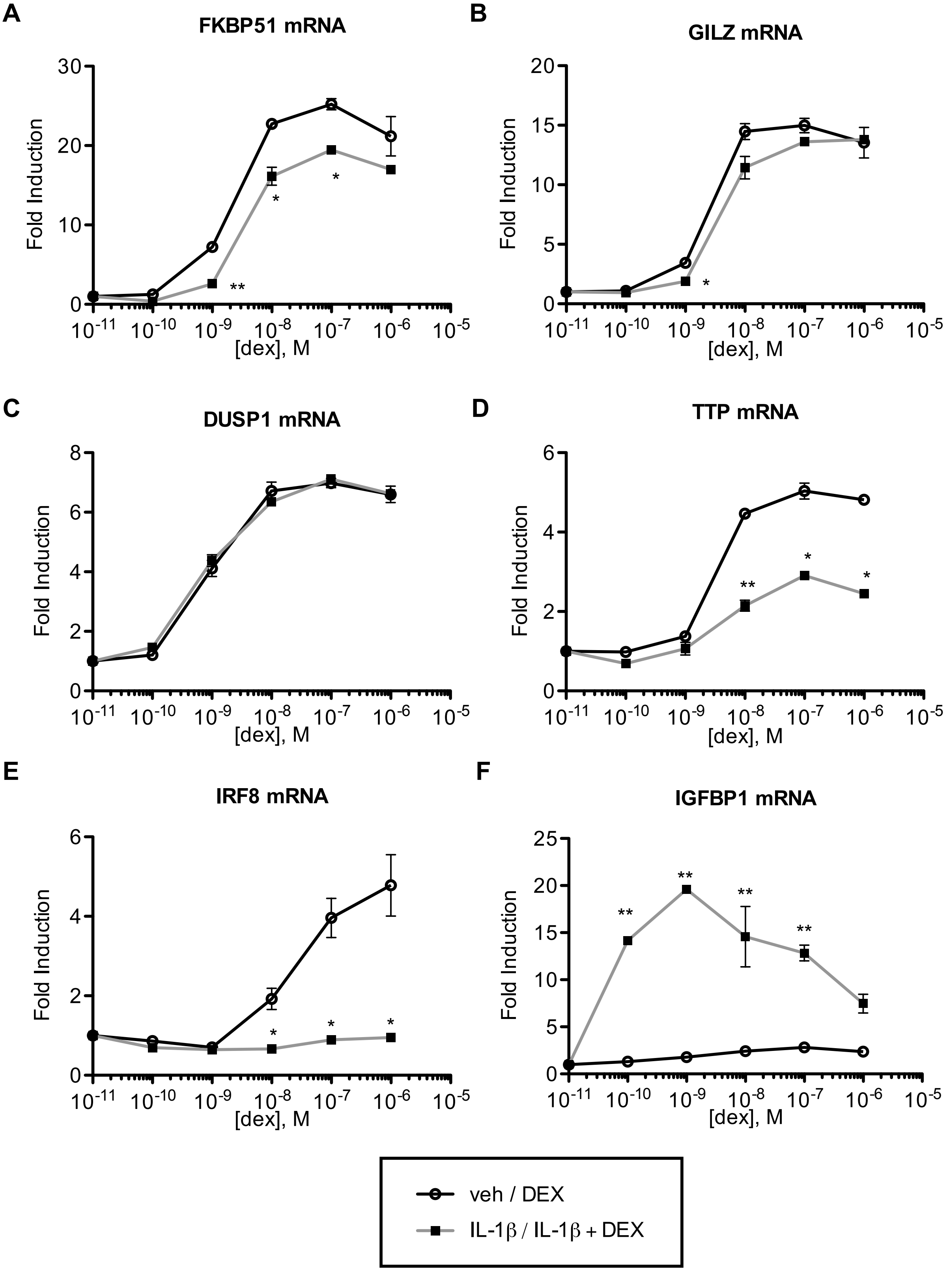

Figure S7. GC dose-response curves of FKBP51, GILZ, DUSP1, TTP, IRF8, and IGFBP1 mRNA expression under DEX alone or sustained IL-1β conditions. Cells were pretreated with 5ng/ml IL-1β or vehicle for 16h and then stimulated 5h with the indicated DEX concentration, either in the absence (O, veh/DEX) or presence of 5ng/ml IL-1β (■, IL-1β/IL-1β+DEX). mRNA expression levels of indicated GC-responsive genes were analyzed by real-time quantitative RT-PCR: (**A**) FKBP51, (**B**) GILZ, (**C**) DUSP1, (**D**) TTP, (**E**) IRF8 and (**F**) IGFBP1. Dose-response curves depict the fold-induction in the expression of the indicated gene for each culture condition compared to 10^{-11} M veh/DEX-treated cells. Data are represented as mean±SD (n = 5). *p<0.05 and **p<0.005 IL-1β/IL-1β+DEX-treated versus veh/DEX-treated cells compared at the same DEX concentration.

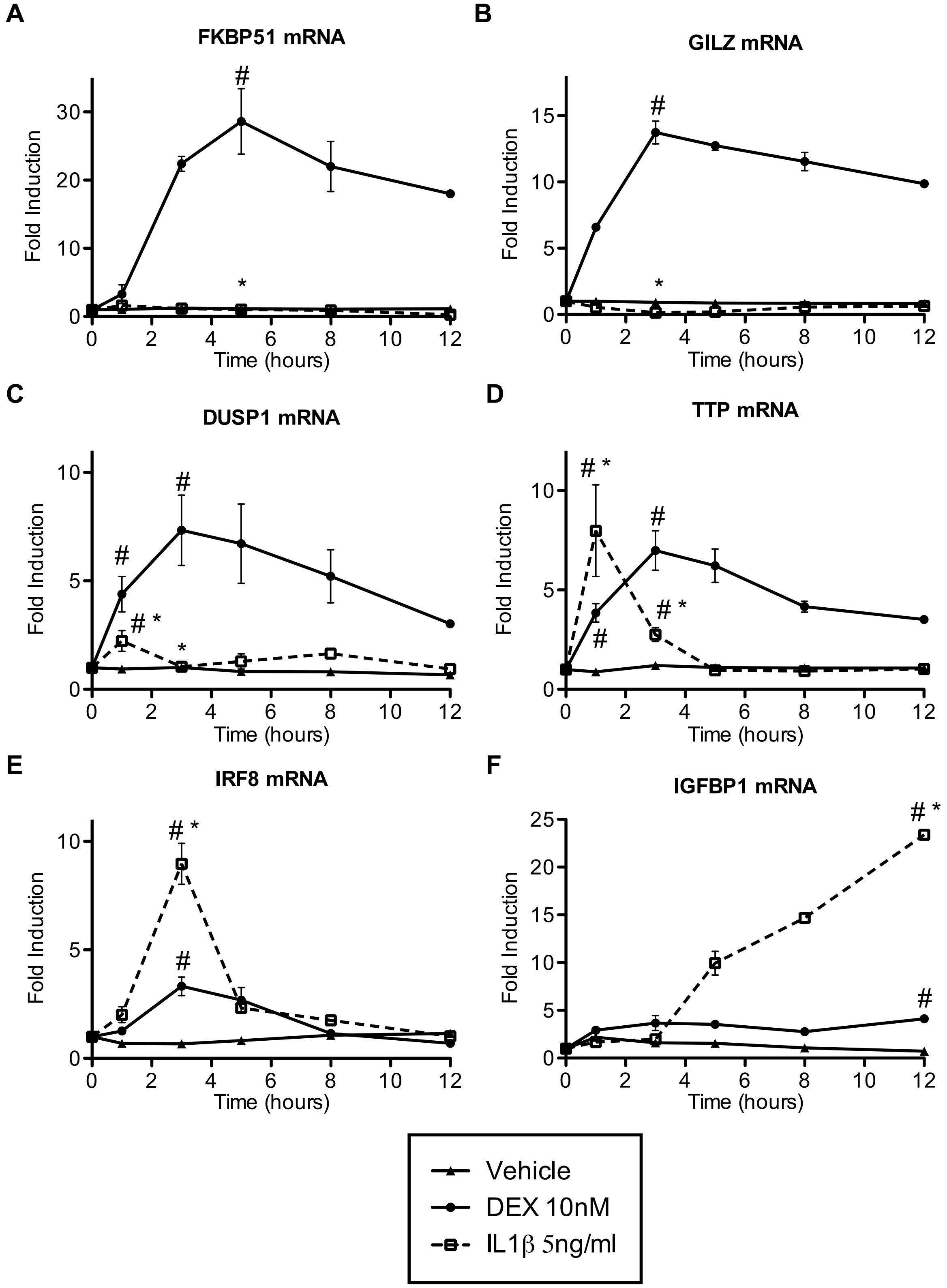

Figure S8. Kinetics of FKBP51, GILZ, DUSP1, TTP, IRF8, and IGFBP1 mRNA expression after IL-1β-or DEX-alone stimulation. Cells were left untreated (veh, ▲, solid line) or stimulated for the indicated times either with 10^{-8} M DEX (10nM DEX, ●, solid line) or 5ng/ml IL-1β (IL-1β, □, dashed line). At the indicated times, the cells were collected, and the mRNA extracted and quantitated by real-time qRT-PCR. For each culture condition, the time-course curves depict the fold-changes in the expression of (**A**) FKBP51, (**B**) GILZ, (**C**) DUSP1, (**D**) TTP, (**E**) IRF8 and (**F**) IGFBP1 compared to vehicle-treated cells at 0h (n = 3). Data are represented as mean±SEM (n = 3). $^{\#}p<0.05$ DEX-treated and IL-1β-treated versus vehicle-treated cells. $^{*}p<0.05$ IL-1β-treated versus DEX-treated cells.

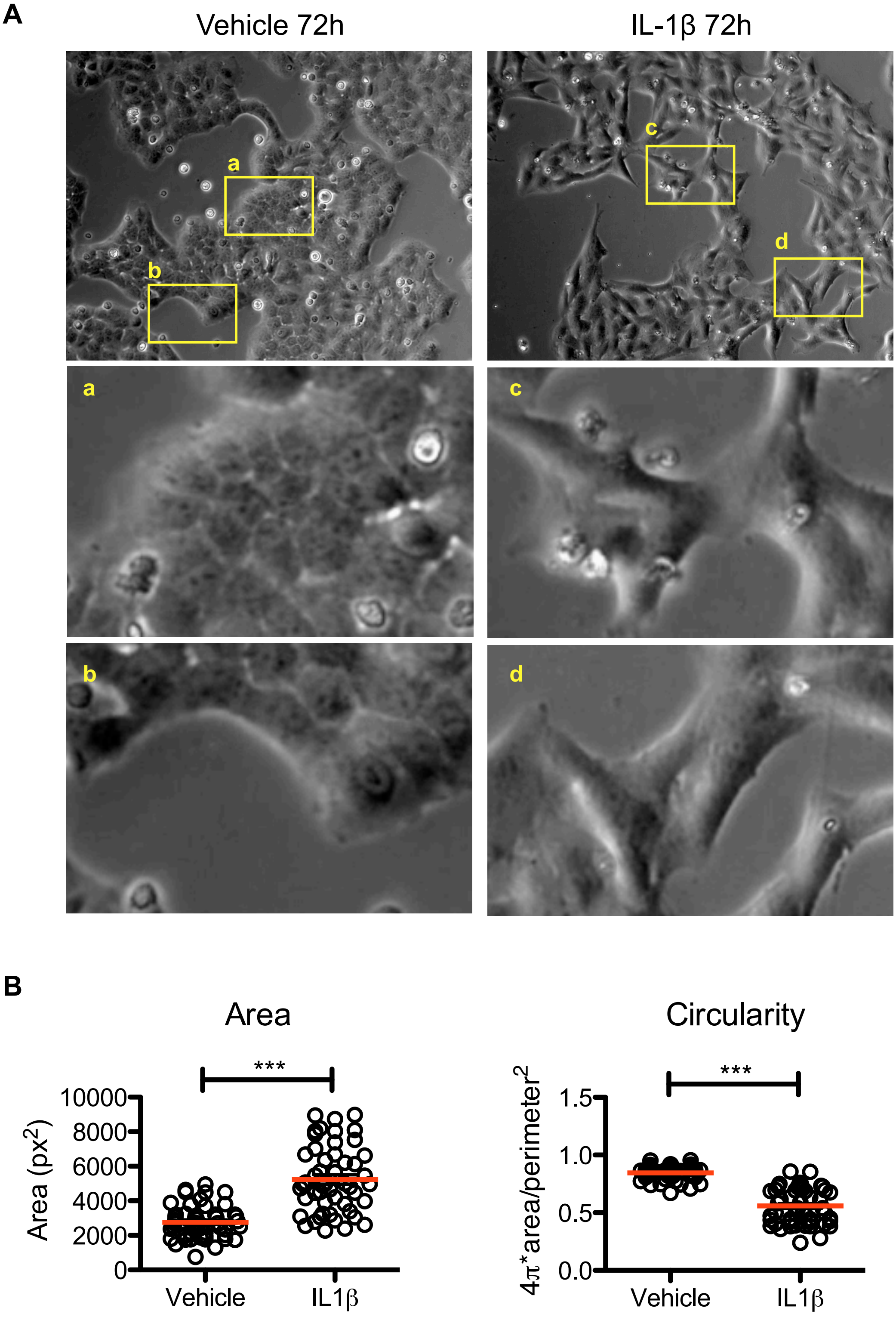

Figure S9. **Prolonged 72h IL-1β exposure promotes changes in the morphological phenotype of A549 cells reminiscent of epithelial-mesenchymal transition (EMT).** Cells were left untreated (vehicle) or treated with 5ng/ml IL-1β (IL-1β) for 72h, and imaged at 20x magnification using phase contrast microscopy. (**A**) Representative fields and the indicated magnification boxes (a-d), for each culture condition. (**B**) Individual area and circularity of 50 cells from 3 different fields for each condition. The red line represents the mean value for each condition. ***p<0.0005 IL-1β-treated versus vehicle-treated cells.
